# A Comprehensive Review of the Nutritional and Health-Promoting Properties of Edible Parts of Selected *Cucurbitaceae* Plants

**DOI:** 10.3390/foods14071200

**Published:** 2025-03-29

**Authors:** Magdalena Borecka, Monika Karaś

**Affiliations:** Department of Biochemistry and Food Chemistry, University of Life Sciences in Lublin, 20–704 Lublin, Poland; magdalena.borecka@up.lublin.pl

**Keywords:** cucurbits, bioactive compounds, health benefits, mineral and phytochemical composition, by-products

## Abstract

The *Cucurbitaceae* family includes commonly consumed plants such as pumpkin, watermelon, melon, horned melon, and cucumber, which are valued for their rich nutritional composition and health-promoting properties. These plants provide essential macronutrients, minerals, and bioactive compounds that contribute to their dietary and therapeutic significance. Particularly, *Cucurbitaceae* plants exhibit antidiabetic, hypolipidemic, antioxidant, and anticancer properties, making them valuable in addressing metabolic disorders and alleviating health risks associated with oxidative stress. This review aims to systematize current knowledge on selected cucurbits’ nutritional composition, mineral content, and phytochemical profile. It also examines their caloric value, glycemic index, and glycemic load, offering insight into their potential role in dietary strategies for patients with obesity, insulin resistance, or diabetes. Additionally, this review explores often-overlooked by-products, including seeds, leaves, and flowers, which are rich in bioactive compounds with potential health benefits. By compiling and analyzing existing data, this review highlights the nutritional and functional potential of *Cucurbitaceae* plants, reinforcing their significance in a health-promoting diet and disease prevention.

## 1. Introduction

The *Cucurbitaceae* family has gained increasing attention in recent years due to the growing global interest in plant-based diets and functional foods. As more people embrace a plant-based approach and look for natural, nutrient-dense foods, cucurbits have become a relevant part of contemporary dietary patterns. The growing popularity of functional foods that provide additional health benefits beyond basic nutrition has further highlighted the potential of cucurbits for disease prevention and health management [1,2]. The *Cucurbitaceae* family is considered one of the most diverse groups. The most popular representatives of this group include pumpkin, watermelon, melon, horned melon known as kiwano, and cucumber [1]. The abundance of vegetable and fruit crops is an important aspect of the national and local economy [2]. The *Cucurbitaceae* family comprises not only edible vegetables and fruits commonly utilized in cooking but also valuable seeds, often regarded as by-products in various industrial processes. These seeds can be consumed directly, such as dehulled pumpkin seeds frequently incorporated into salads. Notably, the high-fat content of *Cucurbitaceae* seeds makes them an excellent resource for vegetable oil production. Additionally, the residual by-products of oil extraction, known as seed cakes, often dismissed as waste, have proven to be rich sources of valuable nutrients and compounds [3,4]. Similarly, the peels of vegetables and fruits from the *Cucurbitaceae* family, though typically overlooked in the food industry, are also rich sources of valuable nutrients. The high content of macro- and microelements, as well as low caloric value, low glycemic index, and load in vegetables and fruits belonging to the group discussed, allows us to state that they should be part of the daily diet, which is an integral element in maintaining human health [1]. The abundance of bioactive compounds found in *Cucurbitaceae* plants contributes significantly to their wide-ranging therapeutic effects. These include vitamins, polyphenols, flavonoids, and essential fatty acids, which contribute to disease prevention and overall health improvement. Such compounds are particularly noted for their anti-inflammatory effects and their potential in managing chronic diseases like diabetes, cardiovascular disorders, and certain cancers, making these plants valuable for both dietary and medicinal purposes [1].

This article aims to review selected representatives of the *Cucurbitaceae* family, focusing on their nutritional value, mineral composition, and the phytochemical compounds responsible for their health-promoting properties.

**Figure 1:**
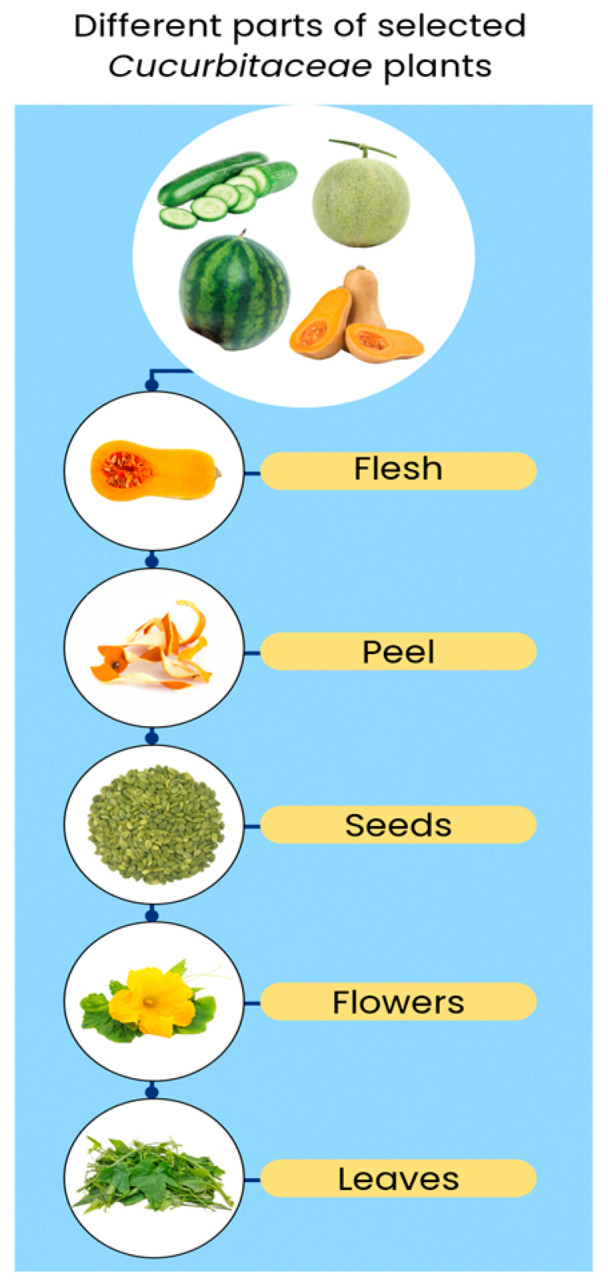
Edible parts of selected *Cucurbitaceae* plants.

This article provides a comprehensive and structured approach to the analysis of the *Cucurbitaceae* family. It combines a detailed description of the nutritional composition and nutritional value of four commonly consumed representatives: pumpkin, watermelon, melon, and cucumber. What distinguishes this review is its broad perspective. Most of the existing reviews primarily focus on fruits and seeds, while this article considers less common parts such as flowers and leaves as well as by-products such as seed cakes and peels (Figure 1). On top of that, the article highlights therapeutic properties indicating potential applications of plants and their bioactive components in various fields beyond the food sector. This insightful look provides an opportunity to understand the full nutritional and health potential of *Cucurbitaceae* in one comprehensive work.

## 2. General Characteristics of *Cucurbitaceae* Family Plants

The plants of the *Cucurbitaceae* family are currently well known and characterized. The family of these plants includes 115 genera and 960 species [2]. Plants from the *Cucurbitaceae* family are among the first cultivated by humans. It is claimed that their cultivation for food purposes began in West Asia as early as 3000 years ago [1]. Their division is based on shape and harvest time, which falls in the summer and winter [4]. Plants belonging to the *Cucurbitaceae* family are considered one of the most diverse groups in terms of cultivation and environmental conditions [1]. These plants are found in tropical and subtropical zones, less frequently in the temperate zone, and are usually monoecious or dioecious, although hermaphrodite plants also occur [2]. The leaves of plants belonging to the *Cucurbitaceae* family can be characterized as thorny, as can the stems of these plants. Additionally, the flowers of these plants are usually yellow and orange, and the fruits that develop from them differ significantly in shape and size [4]. *Cucurbitaceae* include a wide range of vegetables and fruits. The most well-known vegetables in this group include cucumber (*Cucumis sativus*), zucchini, pumpkin (*Cucurbita pepo*, *Cucurbita maxima*, *Cucurbita moschata*, and *Cucurbita argyrosperma*), wax gourd (*Benincasa hispida*), bottle gourd (*Lagenaria siceraria*), bitter gourd (*Momordica charantia*), ridge gourd (*Luffa acutangula*), sponge gourd (*Luffa cylindrica*), chayote (*Sechium edule*), and snake gourd (*Trichosanthes anguina*) and, among fruits, melon (*Cucumis melo*), horned cucumber (*Cucumis metuliferus*), watermelon (*Citrullus lanatus*), and luo-hanguo (*Siraitia grosvenorii*) [2]. It is noteworthy that plants from the cucurbit family are not only valuable for their flavor but also for their therapeutic importance. Mukherjee et al. describe in detail the ethnopharmacological potential of these plants including the therapeutic properties of *Cucumis melo* (musk melon), *Cucumis sativus* (cucumber), *Cucurbita maxima* (pumpkin), *Cucurbita pepo* (field pumpkin), and *Citrullus lanatus* (watermelon) [5].

## 3. Nutritional Value of *Cucurbitaceae* Family Plants

Discussing the nutritional composition of plants from the *Cucurbitaceae* family, it is worth analyzing not only the commonly consumed fruits but also the by-products such as flowers, seeds and oil cake, peels, and leaves of these plants, which serve as valuable sources of nutrients [3,6,7]. The macronutrient profile of cucurbits varies by species, with peels and seeds in general containing higher amounts of proteins, fats, and fiber compared to flesh. In general, most *Cucurbitaceae* plants consist of flesh, peel, and seeds [4,7].

Pumpkins consist of flesh (78.69%), peel (17.95%), and seeds (3.63%) [6]. Comparing the nutritional composition of three Cucurbita species (*C. moschata*, *C. pepo*, and *C. maxima*), significant differences can be observed. The highest carbohydrate content was found in the flesh and peel of *C. pepo* (48.40% and 10.65%, respectively), while the protein and lipid contents were highest in the seeds of the three plants discussed. The highest dietary fiber content was found in the peel of *C. moschata* (33.92%). The moisture content in all three species was similar; however, the highest results were found for *C. pepo* (96.77%) [4]. Generally, it can be assumed that the peel and seeds are richer in nutritional composition compared to the flesh [4]. Watermelon also consists of flesh, which constitutes 40% of its biomass, seeds, and peel, which together constitute about 60% of its mass. Importantly, watermelon seeds and peel constitute a large agricultural waste compared to the by-products of pumpkin (45%), melon (40%), or bitter apple (30%) [8]. Watermelon contains about 91% water; hence, the moisture content in fresh fruit is at the level of 90.82–95.00%. Watermelon flesh is characterized by low protein, lipid, and dietary fiber content (0.12–0.60%, 0.05–0.10%, and 0.20–0.60%, respectively). Among the macronutrients in fresh watermelon fruit, carbohydrates are the most abundant (3.10–8.00%), although this cannot be considered a large amount [9]. The protein content in fresh melon is estimated at 0.5% to 1.31%. The moisture content of the melon peel is relatively low (16.95%), but its ash content is higher than other cucurbits. For instance, the ash content in pumpkin peel is 2.46% and in watermelon peel 3.07%. Additionally, the fat content in melon peel is estimated at 1.58–2.12%, and the dietary fiber content is 41.69% [9]. Kiwano pulp is characterized by high water content (89–96 g/100 g fresh weight). Kiwano peel, in comparison to the pulp, contains significantly more carbohydrates (54.80 vs. 7.56 g/100 g fresh weight), which is similar in the case of fiber, lipids, and protein [10]. Cucumber fruit is characterized by high water content (96.4%) but low carbohydrate content (2.8%), protein, and fat content (0.4% and 0.1%, respectively) [11].

Cucurbit seeds were considered worthless and were by-products of processing. Over the years, it turned out that this part of vegetables and fruits can also be a source of basic macronutrients [7,12]. Pumpkin seeds vary in macronutrient content depending on the species and growing conditions. Generally, it can be assumed that the water content in pumpkin seeds is at the level of 6.37–6.56%. Pumpkin seeds can be a suitable source of fat and protein as they constitute 35–50% of the seed biomass for fat and 25–37% of the seed content for protein. Next are carbohydrates and fiber, which constitute 18–25% and 3–6%, respectively [6]. Another material that is considered a by-product but is valuable in the nutrients discussed is oil cake. Pumpkin oil cake is a by-product obtained during cold pressing of oil from seeds, and it is mainly used as animal feed [13]. The crude protein in pumpkin seed oil cake ranges from 65.30 to 68.90%, while the crude oil content is estimated to range from 8.70 to 10.30% [14]. The number of individual macronutrients in watermelon seeds also varies significantly depending on the species. In general, it can be assumed that watermelon seeds, like pumpkin seeds, are a good source of fats and proteins (24–58% and 17–50% of the seed content, respectively) [8]. Similarly, melon seeds are rich in fats (13–37% of seed content) and proteins (13–37% of their biomass content). Carbohydrate content is at the level of 6–28%, and crude fiber content is at the level of 7–44% of biomass [12].

Kiwano seeds are primarily a source of carbohydrates (50.2 g/100 g fresh weight). The amount of fiber and lipids is at a similar level (19.2 g/100 g fresh weight and 15.4 g/100 g fresh weight, respectively). Of all the nutrients, protein is present in the smallest amount (2.63 g/100 g fresh weight) [10]. Cucumber seeds contain about 95.79% oil and 38.88% protein, with the ash content estimated at 6.48% [15].

Flowers and leaves also provide essential nutrients. The protein content in pumpkin flowers averages 1.31 g/100 g fresh weight, while carbohydrate content is around 3.84 g/100 g [16]. Studies conducted by Mohd et al. [17] on the species *T. occidentalis* established that pumpkin leaves contain 56% protein, 26.82% carbohydrates, and only 2% fats [17,18]. The nutritional composition of selected *Cucurbitaceae* family plants is presented in Table 1.

## 4. Antinutritional Factors and Toxic Constituents in *Cucurbitaceae* Family Plants

Although cucurbits are abundant in numerous nutritional components, *Cucurbitaceae* plants also contain various antinutritional compounds, such as alkaloids, saponins, tannins, oxalates, and cyanogenic glycosides, which can influence nutrient absorption and metabolism. Antinutrients are defined as food constituents that can adversely affect the intake or absorption of essential nutrients by forming toxic compounds during their breakdown. The impact of these ingredients on human health is still under debate. Although there is ample evidence of the positive effects caused by antinutritional ingredients, among others, antioxidant, anti-inflammatory, and antiobesity properties, their negative effects cannot be ruled out. Antinutrients can reduce the bioavailability of other nutrients, including metal ions, amino acids, lipids, and vitamins; hence, it is assumed that their consumption in the long term can lead to health problems such as intestinal integrity [21]. Similarly, in the case of cucurbiticin, which on the one hand has several health-promoting properties, but on the other hand its toxic effects create significant risks associated with the consumption of certain *Cucurbitaceae* plants. Regardless of the toxic effects of cucurbitacins, recent advances have highlighted their application as anticancer agents. Numerous in vitro and in vivo studies demonstrate the capacity of cucurbitacins to inhibit cell proliferation and trigger apoptosis across a wide range of cancer cell lines [22].

Additionally, bitter cucumbers and some varieties of pumpkin, melon, and squash (*Cucumis sativus*, *Cucurbita moschata*, *Cucumis melo*, and *Cucurbita pepo*) can accumulate higher concentrations of cucurbitacins, especially when grown under stress conditions (such as temperature extremes or improper cultivation practices). Additionally, a distinct subgroup of cucurbitacins, known as momordicosides, is present in Momordica charantia (bitter melon or African cucumber) [23]. The consumption of such bitter fruits can be harmful, and it is advised to discard any overly bitter fruits from these plants. It is also essential to note that these compounds are more concentrated in the skin, seeds, and stems of some varieties. Hence, despite its proven therapeutic properties, it is worth taking caution, especially with genetically modified varieties [24].

Various processing techniques, such as fermentation, heat treatment, sprouting, and toasting, can affect the content of antinutritional compounds in different parts of *Cucurbitaceae* plants, potentially reducing their negative effects and increasing the bioavailability of nutrients [21,25,26].

In addition to the potential risks associated with antinutritional compounds and toxic constituents like cucurbitacin, the safety of consuming *Cucurbitaceae* plants is further influenced by microbiological hazards. The consumption of raw Cucurbitaceae plants brings significant microbiological risks associated with contamination by various pathogens. The presence of bacteria, parasites, and viruses can lead to food-borne illnesses, which is a significant danger to human health. Contamination often occurs during cultivation, handling, and processing and is compounded by inadequate hygiene practices and improper storage conditions [27].

## 5. Mineral Composition

Providing minerals through regular consumption of foods with high mineral content is essential for many physiological processes occurring in the body. Plants from the *Cucurbitaceae* family and their various fractions are a good source of minerals and vitamins so it is worth including them in daily diet [7]. The content of individual macro- and microelements varies depending on the species and part of the pumpkin (Table 2).

The peel and fruit of the pumpkin are excellent sources of potassium (687.467 mg/100 g and 340 mg/100 g, respectively). Additionally, the peel is characterized by a high content of sodium and iron (9.652 mg/100 g and 4.004 mg/100 g, respectively) compared to other elements. The pumpkin fruit contains significant amounts of phosphorus, calcium, and magnesium (44 mg/100 g, 21 mg/100 g, and 12 mg/100 g, respectively) [7]. As with pumpkin, the content of individual macronutrients and micronutrients varies depending on the part of the watermelon. The flesh and peel of the watermelon are mostly rich in potassium (18.53 mg/g and 88.67 mg/g). The high content of nitrogen and phosphorus was also noted both in fresh fruit and in the peel [28].

According to Mallek-Ayadi et al. [19], the melon pulp (*Cucumis melo* L.) is characterized by the highest potassium content (2113.75 mg/100 g).

The next in terms of quantity were calcium and magnesium (855.25 mg/100 g and 328.75 mg/100 g, respectively). Among the minerals tested, the lowest value was observed for sodium (137.58 mg/100 g) [19]. Melon peel is characterized by a high potassium content (110.39–1791.9 mg/100 g), as well as a high sodium content (8.54–1770 mg/100 g) and magnesium (0.41–4.1 mg/100 g) [12].

The mineral component that occurs in the largest amount in the kiwano pulp is po-tassium (104–302 mg/100 g fresh weight). The second in order, although occurring in much smaller amounts, are magnesium and calcium [10].

In the case of cucumber fruits, the highest contents were found for potassium, calcium, and magnesium (136 mg/100 g, 14 mg/100 g, and 12 mg/100 g, respectively). Similar to cucumber fruit, the peel also contains the most potassium (454 mg/100 g). High content was also noted for calcium and sodium (168 mg/100 g and 113 mg/100 g, respectively) [20].

The seeds of selected cucurbit plants have long been treated as agro-industrial waste, and it has been discovered that they are a source of minerals valuable for proper functioning [3]. The mineral profile of pumpkins varies depending on the species. Generally, it is assumed that pumpkin seeds are particularly rich in potassium. The content of this element ranges from 237.24 to 982.00 mg/100 g. The second element that occurs in large quantities in pumpkin seeds is phosphorus, the content of which ranges from 47.68 to 1570 mg/100 g. The third element that occurs in significant amounts in pumpkin seeds is magnesium. The content of this element has been established at a level of 283.28 to 2385.00 mg/100 g. Other elements that occur in pumpkin seeds and play key roles in the proper course of physiological processes are zinc (1.24–14.14 mg/100 g), iron (6.66–13.66 mg/100 g), calcium (44.92–141.00 mg/100 g), and sodium (3.54–189.81 mg/100 g). In the context of the mineral profile of pumpkin seeds, it is worth mentioning manganese, selenium, and copper, the contents of which are (0.06–8.90 mg/100 g; 0.13–2.57 mg/100 g; 1.38–1.91 mg/100 g, respectively) [6]. In turn, pumpkin seed oil cake is an excellent source of iron (197.9–198.9 mg/kg dry matter) and zinc (129.4–134.7 mg/kg dry matter). Other elements that occur in significant amounts in oil cake are manganese, copper, phosphorus, and potassium [14].

Watermelon seeds are also characterized by a high content of elements. Similar to pumpkin, watermelon seeds also contain the most potassium of all minerals (3.40–3.5 mg/100 g). Additionally, watermelon seeds contain other minerals such as calcium (0.11–0.16/100 g), phosphorus (0.17–0.22 mg/100 g), magnesium (0.14–0.17 mg/100 g), and zinc (0.66–3.71 mg/100 g) [8,28]. Jaroszewska et al. [29] also examined the mineral profile of watermelon seed cakes. Among the macroelements, the element that occurred in the highest amount was potassium (7.5 g/kg dry matter), while among the microelements, it was iron (124.20 mg/kg dry matter) [29].

Melon seeds, which are also considered by-products, are rich in various minerals. Like pumpkin and watermelon seeds, melon seeds contain large amounts of potassium (509.9–9548.33 mg/100 g). Melon seeds also contain quite a high magnesium content (90.00–3299.27 mg/100 g), as well as sodium (41.22–1770 mg/100 g). Other elements present in smaller amounts in melon seeds include zinc, iron, copper, manganese, and calcium [12]. Potassium is the mineral found in the highest amount in kiwano seeds (1174 mg/100 g fresh weight). The next mineral in terms of quantity in kiwano seeds is magnesium (289 mg/100 g fresh weight) [10]. The mineral profile of cucumber seeds is similar to the seeds discussed earlier. Again, the element that occurs in the highest amount in cucumber seeds is potassium (541 mg/100 g). The second-largest amount is calcium (177 mg/100 g), and the third largest amount is sodium (156 mg/100 g). Other elements found in cucumber seeds in smaller amounts include magnesium, zinc, iron, copper, manganese, lead, cadmium, and nickel [20].

When analyzing the mineral profile of selected plants from the *Cucurbitaceae* family, it is worth paying attention to the leaves and flowers of these plants, which can also be a source of minerals. Based on the research by Mohd et al. [17], who analyzed the mineral composition of the pumpkin leaves of the species *T. occidentalis*, it was found that the leaves contain significant amounts of sodium (89.7 mg/100 g), magnesium (33.0 mg/100 g), calcium (75.0 mg/100 g), potassium (352.0 mg/100 g), copper (36.4 mg/100 g), and zinc (19.3 mg/100 g) [17,18].

Watermelon leaves also stand out in terms of the content of elements. Among the elements that occur in the largest amounts, potassium is listed first (61.10–78.59 mg/g) and then calcium (27.61–61.9 mg/g) and nitrogen (45.06–56.36 mg/g). Among other macro and micro-elements in watermelon leaves are phosphorus (5.76–6.71 mg/g), magnesium (5.26–7.55 mg/g), zinc (0.119–0.18 mg/g), manganese (0.003–0.024 mg/g), and copper (0.003–0.024 mg/g) [28].

Analyzing the mineral profile of the edible flowers of eight pumpkin species, it was found that potassium is also a leading element in this part of the plant, and its value between the species tested ranges from 199.73 to 349.66 mg/100 g. The second-most abundant element is magnesium (17.71–35.83 mg/100 g). Similar values were also noted for calcium (13.78–37.77 mg/100 g). Edible pumpkin flowers also contain small amounts of iron, zinc, and manganese (1.26–5.29 mg/100 g; 0.48–0.78 mg/100 g; 0.18–0.38 mg/100 g) [16].

## 6. Phytochemical Composition

Plants from the *Cucurbitaceae* family are rich in compounds such as carotenoids, phenolic compounds (hydroxycinnamic acids and tocopherols), terpenoids (cucurbitacin), and sterols (avenasterol), which determine their exceptional therapeutic importance [5].

Carotenoids are natural pigments from the tetraterpenoid group, which are soluble in lipids and give plants yellow, orange, and red colors. Pumpkin is characterized by a high content of carotenoids [30]. Among the compounds belonging to this group, which are found in pumpkin, are the following: *α*- and *β*-carotene; *ζ*-carotene; neoxanthin; violaxanthin; lutein; zeaxanthin; taraxanthin; luteoxanthin; auroxanthin; neurosporen; flavoxanthin; 5,6,5′,6′-diepoxy-*β*-carotene; phytofluene; *α*-cryptoxanthin; and *β*-cryptoxanthin. The content of individual compounds varies depending on the species [4]. It is estimated that the total carotenoid content is similar between pumpkin species; however, in *C. pepo* fruits, it can be as high as 461.9 μg/g, while in *C. moschata* fruits, the content is from 171.9 μg/g to 461.9 μg/g [31]. The number of carotenoids in cucurbits may be determined by other factors such as the ripening phase and soil and climate conditions, as well as the processing of harvests and postharvests [32,33]. The ripening period of the fruit is associated with a more remarkable synthesis of carotenoid compounds, which correlates with a high concentration of individual compounds in the plants. Additionally, it was noted that temperature also influences the synthesis of the discussed compounds, and it is lower at low temperatures [30]. Pumpkin fruits are characterized by low phenolic content; for instance, Zakaria Eleiwa et al. [34] did not find flavonoids in *C. moschata* fruits. Also, Yang et al. [35] did not find flavonoid compounds in both unripe and ripe *C. maxima* fruits. *C. pepo* is also considered a poor variety in polyphenols [35]. It was studied that in the case of the aqueous extracts of *C. ficifolia* fruits, the main polyphenolic compounds are *p*-coumaric acid, *p*-hydroxybenzoic acid, salicin, stigmast-7,2,2-dien-3-ol, and stigmast-7-en-3-ol [4,36].

Cucurbitacin is defined as a triterpenoid that is characterized by a bitter taste and is a compound isolated from plants of the *Cucurbitaceae* family. These compounds are divided into 12 groups based on their oxygen functionality at different positions [4]. Pumpkin fruits are not rich in cucurbitacin. So far, the presence of cucurbitacin glycosides C (105 µg/g fresh fruit) and E (438 µg/g fresh fruit) has been detected in *C. pepo* fruits [37]. Other biologically active compounds in pumpkin flesh include functional carbohydrates and polysaccharides. As reported by Xia and Wang [38], the *C. ficifolia* variety (seedless) is rich in D-chiro-inositol, myoinositol, fagopyritols, and sucrose (Table 3).

The carotenoid content affects the color of the flesh in watermelon. Yellow-fleshed watermelons contain negligible amounts (<11 ppm), while red-fleshed varieties can contain up to 15 mg/kg of carotenoids. The red color of the flesh is determined primarily by lycopene and *β*-carotene, while neoxanthin is considered the dominant compound in yellow-fleshed varieties. The percentage of carotenoids in watermelon with red flesh is estimated to be 99%, while in watermelon with yellow flesh, it is only 31%. Red-fleshed watermelons also contain phytofluene, phytoene, γ-carotene, ζ-carotene, and α-carotene but in smaller amounts. The main pigments that are responsible for the orange color of watermelon flesh are *β*-carotene, prolycopene, phytoene, and *ζ*-carotene. The content of individual carotenoid compounds decreases with the ripening of the fruit, which is caused by the decrease in the amount of chlorophyll [39]. Xanthophylls are yellow pigments that are produced by the oxidation of the carotenoids *α*- and *β*-cryptoxanthin, from which lutein and zeaxanthin are produced by the hydroxylation of *α*- and *β*-carotene. The content of xanthophyll varies depending on the color of the watermelon flesh, with the highest content in watermelons with red flesh and the lowest in those with yellow flesh. After analyzing the content of carotenoids at four stages of fruit development and ripening by Grassi et al. [40], it was noted that the number of carotenoids changed at each stage, except for lutein and phytofluene, whose content remained at a constantly low level. It was noted that the compound that occurred at high concentrations at all stages was lutein [39,40]. Other carotenoids found in high concentrations in the red and orange flesh of watermelon include phytoene, phytofluene, *γ*-carotene, and *ζ*-carotene. These compounds are also precursors in the biosynthesis of lycopene [39]. The phenolic content of watermelon varies significantly depending on its parts (pulp, peel, seeds, and leaves), but the total phenolic content is higher in the ring than in the pulp, and the total polyphenol content ranges from 137 to 260 mg depending on the variety [39,41]. Among the non-flavonoid phenolic compounds in the watermelon pulp are 11-hydroxybenzoic acid and 20-hydroxycinnamic acid derivatives, a significant part of which is combined with sugars. Additionally, in this part of the watermelon, the presence of coumarin derivatives such as coumarin, aviprin, and obtusoside has been found [39]. Watermelon is also a natural source of L-citrulline, which in the urea cycle is a precursor of arginine-an amino acid necessary for proper blood flow and the associated level of nitric oxide. The content of L-citrulline varies depending on the variety and color of the flesh. Watermelons with yellow and orange flesh are characterized by a higher level of the discussed amino acid (25.5 and 14.2 mg/g dry weight, respectively). Among other biologically active compounds isolated from watermelon flesh are cucurbitacin B, C, D, E, I, and cucurbitacin L 2-*O*-b-glucoside [39] (Table 3).

Das et al. studied the content of phytochemical compounds in melon fruits (*C. melo*). The analysis showed the presence of saponins, tannins, phenolic compounds, and terpenoids [42]. The research conducted by Šeregelj et al. [43] found that the total phenolic content in the pulp of horned melon ranges from 47.2 to 200 mg GAE/100 g dry weight, depending on the type of extract. Based on the studies conducted on the extracts of *C. metuliferus* melon pulp by Šovljanski et al. [44], the total carotenoid content was found to be at the level of 0.81–1.14 mg β-car/100 g. The total phenolic content in the tested extracts amounted to 58.22–74.90 mg GAE/100 g, while the content of chlorophylls was estimated in the range of 4.23–5.06 mg/100 g. In all the tested samples, a smaller amount of chlorophyll a was observed than chlorophyll b (~1.41 mg/100 g and ~2.78 mg/100 g, respectively) [44]. Additionally, Ferrara [45] reports that the pulp of horned melon contains lower levels of flavonoid aglycones (myricetin and quercetin) as opposed to flavonol glycosides (rutin), which are present in high concentrations [43] (Table 3).

The compounds that occur in the largest amounts in aqueous extracts of cucumber pulp include carbohydrates, flavonoids, glycosides, steroids, and tannins. The content of carotenoids in cucumber fruits is strictly dependent on the cultivation conditions [46]. Based on studies conducted on two cucumber species (*C. sativus Linn.* and *C. trigonus Roxb.*), it can be assumed that they are a good source of flavonoids, the content of which is 2.63 mg/kg; additionally, a high content of alkaloids (0.82 mg/kg) was found in the tested fruits, compared to the content of tannins, serpentines, lignins, glycosides, terpenoids, and saponins. The total content of phenols was 0.18 mg/kg [47] (Table 3).

**Table 3:** Phytochemicals composition of selected *Cucurbitaceae* plants.

Plant/Part		Bioactive Compounds	References
**Cucumber** **(*C. sativus Linn., C. trigonus Roxb*.)**	fruits	flavonoids (rutin, quercetin, apigenin, kaempferol) (2.63 mg/kg); phenolics TPC (0.18 mg/kg); carotenoids; alkaloids (0.82 mg/kg); tannins; lignins, saponins; glycosides; terpenoids; cucurbitacins (B, C, E), carbohydrates; steroids; serpentines	[9,35,36,37]
	peel	flavonoids (14.02 mg QE/g); tannins; saponins; glycosides; anthraquinones; phenolics (23.08 mg GAE/g)	[48,49]
	seeds	phenolics (93.5 mg/GAE/g); flavonoids (57.4 mg QE/g); β-carotene (19.46 mg/100 g); tannins; terpenoids; saponins; flavonoids	[16,50,51]
	leaves	coumarin A and B; vitexin; isovitexin; orientin; isoorientin; hydroxycinnamic acid	[52]
**Melon** **(*C. melo, C. metuliferus*)**	pulp	saponins; tannins; phenolic compounds (47.2–200 mg GAE/100 g); terpenoids; carotenoids (0.81–1.14 mg β-car/100 g) (lycopene, β-carotene, lutein, phytofluene, phytoene, γ-carotene, ζ-carotene); chlorophylls (4.23–5.06 mg/100 g); flavonoid aglycones (myricetin and quercetin); flavonol glycosides (rutin).	[42,43,44,45]
	peel	3-hydroxybenzoic acid (33.5 mg/100 g); apigenin-7-glycoside (29.3 mg/100 g); isovanilic acid (23.7 mg/100 g); m-coumaric acid; oleuropein; luteolin-7-glycoside; flavonoids (95.5 QE/100 g); β-carotene (821.5 μg/100 g); lycopene (64.5 μg/100 g); glycosides (2.19 mg/g); tannins (1.38 mg/g)	[12,41,53,54,55]
	seeds	alkaloids (2.54 mg/g); steroids (2.62 mg/g); terpenoids; tanins (2.93 mg/g); glycosides; phenolics TPC (29.39 mg/100 g); flavonoids TFC (20.67 mg/100 g); carotenoids (1.56–130 mg/g); α-tocopherol; γ-tocopherol; sitosterol (3248.48 mg/kg oil); Δ 5-avenasterol (1533.11 mg/kg oil); ferulic acid (134.93 µg/g); vanillic acid (2.31 µg/g oil); caffeic acid (1.98 µg/g oil); oleuropein; pinoresinol	[43,56,57]
	leafs	TPC (26.40 mg GAE/g) and TFC (69.7 μg RE/g)	[9,58]
**Pumpkin** **(*C. pepo, C. moschata, C. maxima, C. ficifolia*)**	flesh	carotenoids (171.9–461.9 μg/g) (α- and β-carotene; ζ-carotene; neoxanthin; violaxanthin; lutein; zeaxanthin; taraxanthin; luteoxanthin; auroxanthin; neurosporen; flavoxanthin; 5,6,5′,6′-diepoxy-β-carotene; phytofluene; α-cryptoxanthin; β-cryptoxanthin, tocopherols); phytosterols (β-sitosterol, stigmasterol, campesterol, Δ5-avenasterol); polysaccharides; D-chiro-inositol; myo-inositol; cucurbitacins (C and E) (105 µg/g and 438 µg/g); polyphenolic compounds: p-coumaric acid; p-hydroxybenzoic acid; salicin; stigmast-7,2,2-dien-3-ol; stigmast-7-en-3-ol; carbohydrates: D-chiro-inositol; myoinositol; fagopyritols; sucrose	[4,30,31,36,37,38]
	peel	flavonoids (0.41 mg CE/100 g) and phenolic compounds (1.83 mg GAE/100 g); carotenoid compounds: β-carotene (39.48–123.19 mg/kg)	[4,30,59]
	seeds	phytosterols (265 mg/100 g); squalene; β-sitosterol (0.024–0.038 mg/100 g); stigmasterol (8.40–13.28 mg/100 g); α-tocopherol (0.18–9.66 mg/100 g); β-tocopherol (0.058–1.68 mg/100 g); γ-tocopherol (1.21–62.00 mg/100 g); kaempferol; p-coumaric acid (5.04 mg/kg); ferulic acid; apigenin; quercetin; vanillic acid (9.54 mg/kg); p-hydroxybenzoic acid (27.00 mg/kg); lutein; sinapic acid; tyrosol; vanillin; protocatechuic acid (1.58 mg/kg); fatty acids; primarily oleic; linoleic acids; Δ7-sterols (avenasterol, spinasterol); Δ5-sterol (sitosterol, stigmasterol); campesterol; cempestanol; triterpenoids; sesquiterpenoids; tetraterpenoids (carotenoids); tocopherols	[6,13,60,61,62,63,64,65,66,67]
	flowers	carotenoids (29.8 mg/100 g); phenolics (133.26 mg CAE/100 g; 8.09–17.39 µg GAE/mL); flavonoids (0.51–8.23 mg QE/100 g; 2.29–17.134 QE µg/mL)	[16,68,69]
	leaves	saponins; tannins; alkaloids; flavonoids; glycosides	[18,63,67]
**Watermelon** **(*C. lanatus*)**	flesh	carotenoids in yellow-fleshed varities: neoxanthin; carotenoids in red-fleshed varities: lycopene, β-carotene, phytofluene, phytoene, γ-carotene, ζ-carotene, and α-carotene (15 mg/kg); lutein; zeaxanthin; phenolics (47.3 TAE/g); non-flavonoid phenolic compounds: 11-hydroxybenzoic acid; 20-hydroxycinnamic acid derivatives; coumarin derivatives: coumarin, aviprin, and obtusoside; L-citrulline (25.5 and 14.2 mg/g); cucurbitacin B, C, D, E, I; cucurbitacin L 2- O–b -glucoside	[39,40,41]
	peel	polyphenols (63.33 mg TAE/g); alkaloids; phytates; tannins; oxalate; saponins	[8]
	seeds	phenolics (0.087 mg GAE/g); sinapic acid (152.300 µg/mL); ferulic acid (68.285 µg/mL); 4-hydroxybenzoic acid (59.707 µg/mL); caffeic acid (1.33 μg/g); pinoresinol lignans (and 1.02 μg/g); alkaloids (28.33 mg/g); saponins (16.87 mg/g); flavonoids (0.75 mg/g); alkaloids; tannins; terpenoids; sitosterol (2298.83 mg/kg oil); Δ 5-avenasterol (1319.21 mg/kg seed oil); in seed cakes TFC (721.09 mg); luteolin mg/kg; TPC (1.67 mg GAE/g)	[8,29,48,56,70,71,72]

Additionally, in another study conducted by Oboh et al. [73], the presence of several compounds belonging to the flavonoid group was determined, i.e., rutin, quercetin, apigenin, and kaempferol [9,73]. Cucumber fruits are also rich in compounds belonging to the numerous groups of cucurbitacins, of which the most characteristic for cucumbers are cucurbitacins B, C, and E [9] (Table 3).

The presence of both flavonoids and phenolic compounds was noted in methanol and ethanol extracts of pumpkin peel. According to Asif et al., the TPC (total phenolic content) and TFC (total flavonoid content) value in 80% methanol extract of pumpkin peel was 1.83 mg GAE/100 g and 0.41 mg CE/100 g, respectively [59]. Based on the studies of pumpkin peel powder, it was possible to determine that aspartic acid is the most abundant amino acid found in this part of the pumpkin (2.64%). Additionally, high content was found for glutamic acid and leucine (2.53% and 1.21%, respectively) [74,75]. Pumpkin peel is richer in carotenoid compounds than the flesh. It is estimated that the total carotenoid content of the peel of *C. moschata* is significantly higher than that of the flesh [4]. Moreover, it was found that the peels contained more *β*-carotene than other parts of the three pumpkin species tested (*C. pepo*, *C. moschata*, and *C. maxima*), and these values ranged from 39.48 to 123.19 mg/kg, depending on the species [30]. Similar to pumpkin peel, watermelon peel is also richer in individual bioactive compounds than the pulp. An example of this is polyphenols, the content of which in the peel is at the level of (63.33 ± 1.455 mg TAE/g) and in the watermelon pulp at the level of 47.3 ± 0.88 mg TAE/g. In addition, a higher content in the peel than in the pulp was found for phenols (0.026 ± 0.003 mg GAE/g and 0.010 ± 0.001 mg GAE/g, respectively). The exceptions are flavonoids, the larger amounts of which occur in the pulp. Other functional compounds present in watermelon peel include alkaloids, phytates, tannins, oxalate, and saponins [8]. Based on the research of Mallek-Ayadi et al. [53], nine classes of phenolic compounds were identified in melon peel (*Cucumis melo* L.), among which the highest values were found for 3-hydroxybenzoic acid (33.5 ± 0.37 mg/100 g), apigenin-7-glycoside (29.3 ± 0.17 mg/100 g), and isovanilic acid (23.7 ± 0.04 mg/100 g). Additionally, as reported by the authors, *m*-coumaric acid, oleuropein, and luteolin-7-glycoside also occurred in significant amounts. The content of flavonoids in melon peel is 95.5 ± 0.15 mg QE/100 g of extract [41]. In the group of phytosterols, the compound that occurs in the largest amounts is simiarenol. Based on the studies conducted by Sabino et al. [54] on melon peel flour, the content of *β*-carotene and lycopene was determined (821.5 ± 0.1 μg/100 g and 64.5 ± 0.0 μg/100 g, respectively) [12,54]. Analysis of melon peel (*C. metuliferus*) proved that other compounds that are abundant in this part of the plant are glycosides (2.19 mg/g) and tannins (1.38 mg/g) [55]. Screening of phytochemical compounds carried out by Adamu et al. [48] using extracts from cucumber peel (*C. sativus*) revealed that the compounds that occur in the largest amounts in this part of the cucumber are flavonoids. Among other compounds that are found in the peel but occur in smaller amounts are tannins, saponins, cardiac glycosides, and anthraquinones. In the conducted study, no alkaloids were detected in cucumber peel extracts [48]. The total phenolic and flavonoid contents of ethanolic cucumber peel extracts were determined at 23.08 mg GAE/g and 14.02 mg QE/g, respectively [49] (Table 3).

Pumpkin seeds are rich in many compounds with biological activity, including phytosterols, tocopherols, and squalene [76]. The content of *β*-sitosterol in different varieties of pumpkin is estimated at 0.024–0.038 mg/100 g. Stigmasterol is also a compound that is found in large quantities in pumpkin seeds, and its amount is 8.40–13.28 mg/100 g. As reported by researchers, campesterol, Δ 5-avenasterol, sitostanol, and cempestanol are also present in the seeds [60,61]. The total content of phytosterols in pumpkin seeds is 265 mg/100 g [6,62,77]. Pumpkin seeds are also characterized by a high content of tocopherol fractions. Based on studies conducted on seeds of the *C. pepo*, *C. maxima*, and *C. moschata* varieties, the content of α-tocopherol was determined to be 0.18–9.66 mg/100 g and the content of *β*-tocopherol in the range of 0.058–1.68 mg/100 g [6,63]. The most abundant fraction determined in different pumpkin varieties is *γ*-tocopherol, the content of which is in the range of 1.21–62.00 mg/100 g [6]. Pumpkin seeds are also an excellent source of various phenolic compounds, including kaempferol, *p*-coumaric acid, ferulic acid, apigenin, quercetin, vanillic acid, and *p*-hydroxybenzoic acid. The TPC in pumpkin seeds ranges from 0.97 to 8.27 GAE/g [6]. Pumpkin seed oil is rich in numerous bioactive compounds such as fatty acids and primarily oleic and linoleic acids. Moreover, Δ7-sterols (avenasterol and spinasterol) and Δ5-sterol (sitosterol and stigmasterol) are also present. Furthermore, the oil is characterized by triterpenoids, sesquiterpenoids, tetraterpenoids (carotenoids), tocopherols, and polyphenols. Pumpkin oil also contains significant amounts of squalene, which provides oxidative stability [64]. The TPC in pumpkin seed oil ranges from 24.70 to 50.90 mg GAE/kg oil, and the most abundant compounds are lutein, sinapic acid, tyrosol, vanillic acid, and vanillin [65]. Among the polyunsaturated acids that occur in the largest amounts in pumpkin seed oil are linoleic and oleic acids, the content of which is estimated at 76% of all fatty acids [66,67]. Among tocopherols, the *α-* and *γ*-tocopherol occur in larger quantities than β-tocopherol [62]. The total phenolic acid content in pumpkin seed oil cake in freeform according to Peričin et al. [13] was determined at the level of 43.2 mg/kg dry weight. *P*-Hydroxybenzoic acid was the most abundant of all phenolic acids tested (27.00 mg/kg dry matter). Among other phenolic acids that were discovered in pumpkin seed oil cake are gallic acid 5.4–26.0 (mg/100 g dry weight), protocatechuic acid 5.2–12.9 (mg/100 g dry weight), and vanillic acid 4.71–10.7 (mg/100 g dry weight). Other compounds found in oil seed cakes included trans-*p*-coumaric acid and protocatechuic acid (9.54 mg/kg dry matter; 5.04 mg/kg dry matter and 1.58 mg/kg dry matter, respectively) [13,78].

Watermelon seeds are also rich in some compounds with biological activity. Watermelon seeds are characterized by a high content of phenols. The TPC in the seeds (0.087 mg GAE/g) was higher in this part of the plant compared to the peel and flesh [8]. Fadimu et al. [70] analyzed watermelon seed extracts using an ultrasound-assisted extraction method. Analysis of the extracts for individual phenolic acids showed that sinapic acid was found in the highest concentration (152.300 µg/mL). Among the other compounds that were discovered and whose concentrations were high are ferulic acid and 4-hydroxybenzoic acid (68.285 µg/mL and 59.707 µg/mL, respectively) [70]. Other researchers revealed that other compounds found in watermelon seed are caffeic acid and pinoresinol lignans (1.33 μg/g and 1.02 μg/g, respectively) [56]. Among other phytochemical compounds, high contents of alkaloids and saponins were found in watermelon seeds (28.33 mg/g and 16.87 mg/g, respectively). Flavonoids are present in watermelon seeds in the lowest concentration (0.75 mg/g) [71]. Rekha et al. [72], in their studies on watermelon seed oil, found the presence of alkaloids, saponins, tannins, phenols, and terpenoids. The presence of flavonoids, glycosides, and sterols was not confirmed in the oil [72]. Rezig et al.’s [56] study showed that watermelon seed oil is rich in numerous phytosterols. Among the compounds that occurred in the largest amounts were sitosterol (2298.83 mg/kg oil) and Δ 5-avenasterol (1319.21 mg/kg seed oil). Additionally, phenolic compounds represented by caffeic acid (1.33 µg/g) and pinoresinol (1.02 µg/g) were identified in watermelon seed oil [56]. Jaroszewska et al. [29] also examined the phytochemical composition of watermelon seed cakes. It was determined that the TFC in the tested material was 721.09 mg luteolin mg/kg, while the TPC was 1.67 mg GAE/g [29] (Table 3).

Olubuni et al. analyzed the content of phytochemical compounds in melon (*C. melo*) seeds. The analysis confirmed the presence of secondary metabolites in melon seeds, including alkaloids, phenolics, steroids, flavonoids, terpenoids, and glycosides. The TPC of aqueous extracts of melon seeds was 29.39 mg/100 g, and the TFC was 20.67 mg/100 g [57]. Horned melon seeds are also a reservoir of phytochemicals. Kiwano seeds contain large amounts of tannins, steroids, and alkaloids (2.93 mg/g dry weight, 2.62 mg/g dry weight, and 2.54 mg/g dry weight, respectively). The content of carotenoids is estimated to range from 1.56 to 130 mg/g dry weight. In the group of tocopherols, *α*-tocopherol and γ-tocopherol were identified in the seeds. The TPC is up to 400 mg/GAE/100 g dry weight [43]. Melon seed oils, similar to watermelon seed oil, contain large amounts of sitosterol (3248.48 mg/kg oil), as well as Δ 5-avenasterol (1533.11 mg/kg oil). In addition, among the representatives of phenolic compounds, ferulic acid occurs in the largest amounts (134.93 µg/g). The presence of vanillic acid and caffeic acid (2.31 and 1.98 µg/g) was also detected. Oleuropein and pinoresinol also occur in melon seed oil [72].

Cucumber seeds are characterized by a high content of phenols (93.5 mg/GAE/g), flavonoids (57.4 mg QE/g), and *β*-carotene (19.46 mg carotenoids/100 g) [50]. Furthermore, Achikanu et al. [51] showed that tannins were found in the highest amounts in cucumber seeds (2.934 mg/g seed powder), whereas glycosides and terpenoids were not identified. In the study conducted by Bieżanowska-Kopeć et al. [16], cucumber seed oil was obtained using acetone and ethanol. Two extracts contained large amounts of terpenoids, and both extracts included saponins and flavonoids. The extracts did not contain alkaloids, glycosides, or phenols [51] (Table 3).

Edible pumpkin flowers are also a source of various phytochemicals. Bieżanowska-Kopeć et al. [16] analyzed eight varieties of edible pumpkin flowers and determined that the average content of carotenoids in the fresh weight of flowers was 29.8 mg/100 g, and the TPC was 133.26 mg CAE/100 g. Zhou et al. [68] also analyzed the compounds contained in the flowers of three pumpkin varieties (*C. maxima*, *C. pepo*, and *C. moschata*). The TFC varied between the species, with the highest value recorded for *C. maxima* (8.23 mg QE/100 g fresh weight), while the lowest value was recorded for *C. pepo* (0.51 mg QE/100 g fresh weight) [68]. Ghosh and Rana [69] also studied the phytochemical content of pumpkin (*C. maxima*) flowers. The TPC varied depending on the type of extract. For the aqueous extract, the TPC was 8.09 µg GAE/mL, whereas for the methanolic extract, it was 17.39 µg GAE/mL. The TFC in the aqueous extract was 17.134 QE µg/mL, while the alkaloid content in terms of atropine equivalent was higher for the aqueous extract (2.29 µg/mL) [69] (Table 3).

Pumpkin leaves also constitute a reservoir of bioactive compounds [18]. Muhammed et al. [79] studied the content of phytochemicals in pumpkin leaves (*T. occidentalis*) in ethanolic and aqueous extracts. The authors found the presence of saponins, tannins, alkaloids, and flavonoids in the two extracts studied. The presence of glycosides was confirmed only in the ethanolic extract [79] (Table 3).

There are also bioactive compounds in melon leaves and roots [9]. Ismail et al. [58] examined methanol extracts of different parts of melon for their phytochemical content. The TPC (26.40 mg GAE/g extract) and TFC (69.7 μg RE/g extract) in leaves were the highest among all the parts of melon examined (seeds, flesh, skin, and stem). Interestingly, the stem was the second highest in terms of the TPC and TFC (10.25 mg GAE/g extract and 9.68 μg RE/g extract, respectively) [58] (Table 3).

Cucumber leaves can also be a source of bioactive compounds. Cucumarin A and B, classified as C-glycosides, have been identified in cucumber leaves, as well as vitexin, isovitexin, orientin, isoorientin, and hydroxycinnamic acid [52]. The analysis of pumpkin flowers for phenolic compounds revealed the presence of quercetin-3-*O*-glucoside, kaempferol 3-*O*-glucoside, isorhamnetin 3-*O*-glucoside, and kaempferol 3-*O*-rhamnoside [9,80].

## 7. Caloric Value, Glycemic Index, and Glycemic Load

Obesity and carbohydrate metabolism disorders including type 2 diabetes are global health problems that have been growing in recent years and are associated with an increased incidence of chronic non-communicable diseases but also mortality; hence, it is so important to control the diet in terms of energy value and monosaccharide content [1].

**Figure 2:**
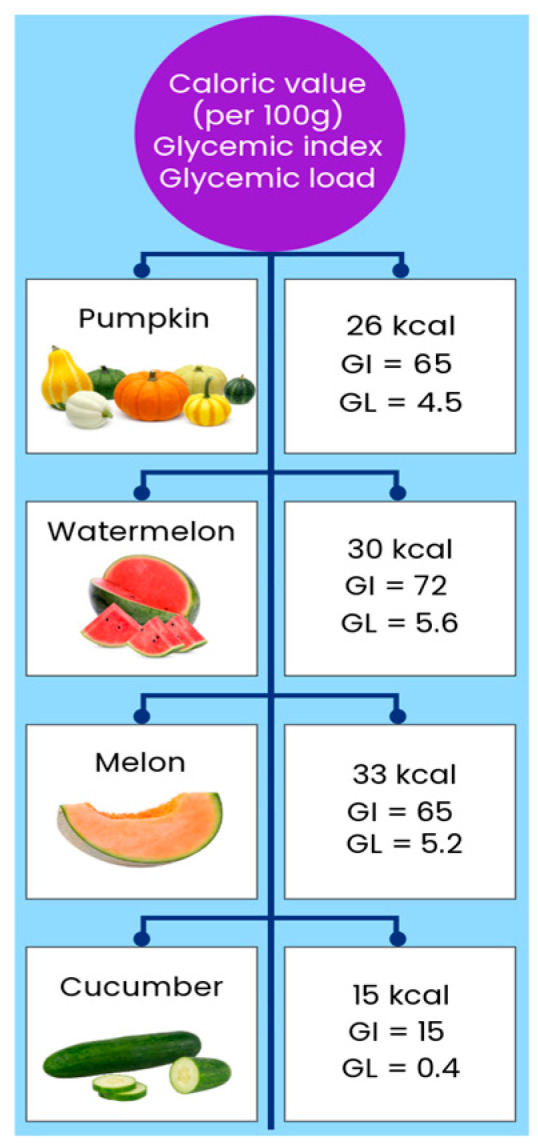
The caloric value of selected *Cucurbitaceae* plants.

*Cucurbitaceae* plants are commonly used in the diet not only because of their high content of various bioactive compounds but also because of their low caloric value as well as low glycemic index (GI) and glycemic load (GL) (Figure 2).

The energy value of selected vegetables and fruits from the *Cucurbitaceae* family is similar. Cucumber (15 kcal/100 g) and zucchini (16 kcal/100 g) have the lowest calorie value. Among fruits, watermelon and melon also have a similar energy value (30 kcal/100 g and 33 kcal/100 g, respectively [81]. GI is defined as the increase in the blood glucose concentration following eating, expressed as the incremental area under the blood glucose curve over two hours. In other words, GI is a scale that ranks a carbohydrate-containing food or drink by how much it raises blood sugar levels after it is eaten or drank. Foods with a high GI increase blood sugar higher and faster than foods with a low GI. Products are divided into low GI (GI ≤ 55), medium GI (GI 56–69), and high GI (GI ≥ 70) [82]. The glycemic response is defined as the appearance of glucose in the blood after consuming food. The glycemic response is largely determined by the amount of food consumed; hence, GL was introduced as another criterion for predicting the glycemic response. GL considers both the GI and the amount of available carbohydrates in a portion of the consumed product (GL = GI × available carbohydrates in a given amount of food). Based on GL, products are divided into those with low GL (GL ≤ 10), medium (GL 11–19), and high (GL ≥ 20) [82]. The vegetables and fruits discussed in the *Cucurbitaceae* family are characterized by a low and medium GI. The exception is watermelon, whose GI is 72. All the vegetables and fruits discussed are classified as having a low GL, with cucumber having the lowest value [81,83].

## 8. Health Properties of *Cucurbitaceae* Family Plants

Plants from the *Cucurbitaceae* family contain bioactive compounds with various health benefits, including lowering lipid and glucose levels, providing antioxidant, anti-inflammatory effects, and potentially aiding in cancer prevention (Table 4).

### 8.1. Hypoglycemic and Antidiabetic Effect

Several studies have been conducted to investigate the antidiabetic properties of plants from the *Cucurbitaceae* family [5]. Hashem Dabaghian et al. [84] found that polysaccharide combined with the isolated protein from *C. pepo* exhibited antidiabetic effects by increasing insulin levels, lowering blood glucose levels, and improving glucose tolerance in diabetic-induced in-vivo models. Moreover, another study investigated the antidiabetic properties of ethanolic extracts of *C. pepo* peels. The study confirmed the improvement of blood glucose and serum lipid in alloxan-induced diabetic rats [5,84]. Compounds contained in pumpkin seeds also exhibit hypoglycemic properties. One of these compounds is tocopherols. Bharti et al. [85] studied the effect of tocopherols extracted from pumpkin seeds in diabetic rats in an in vivo model. The researchers found that after the rats were given the extracts, a decrease in glucose levels was noted [6,85]. Marbun et al. [86] showed that ethanol extracts of pumpkin flesh and seeds (dose level 150 mg/kg) positively lowered blood glucose levels in mice with streptozotocin-induced diabetes. The hypoglycemic effect was comparable to that induced by metformin (65 mg/kg) [86,87].

**Table 4:** The bioactivities of selected *Cucurbitaceae* family plants.

Plants	Part of Plant	Biological Activity	References
**Pumpkin**	Seed oil	anticancer	[7]
	Seeds	antioxidant	[29,88]
**Pumpkin** (*C. maxima*)	Seed (ethanolic extracts)	antidiabetic	[89]
**Pumpkin** (*C. moschata*)	Flesh + seeds (extract)	anticancer	[90]
**Pumpkin** (*C. pepo*)	Flesh + wheat flour	hypolipidemic	[91]
	Seeds (hydroethanolic extract)	anticancer	[3,92]
	Leaf (extracts)	antidiabetic	[18]
	Seed oil	antidiabetic and hypolipidemic	[18,93]
	Peels (ethanolic extracts)	antidiabetic	[84]
			[5]
	Seeds (tocopherols extract)		[85]
	Seeds (extract)	hypolipidemic	[94]
**Watermelon**	Pulp, Seeds, Rind (juice)	antihyperglycemic	[8,95]
**Yellow** **Watermelon**	Flesh	antidiabetic	[96]
**Watermelon** (*C. lanatus*)	Seeds (ethanolic extract)	cardioprotective	[97]
	Seeds (peptides)	antioxidant	[39,98,99]
	Peel extracts	anticancer and antioxidant	[100,101]
**Melon**	Seeds (hexane extract)	antidiabetic	[102]
	Fruits	antioxidant	[103]
	Pulp, seeds, peels		[104]
	Seed and leaf (extract)		[58]
	Methanolic extracts	anticancer	[105]
**Melon** (*C. melo* L.)	Concentrates	cardioprotective	[106]
**Horned Melon** (*Cucumis* metuliferus)	fruit	antiulcer, anti-inflammatory	[43]
	peel	antioxidant and antifungal	[44]
**Cucumber** (*C. sativus*)	Fruits (ethanolic extracts)	antihyperglycemic	[107]
	Seeds (extract)	hypocholesterolemic	[108]
	Peel (ethanol extract)	antioxidant	[49]
	Pulp, Leaf		[109,110]
	Flowers (acetyl extracts)	anticancer	[9]
	Flowers (molecular docking)	antiangiogenic	[111]

**Figure 3:**
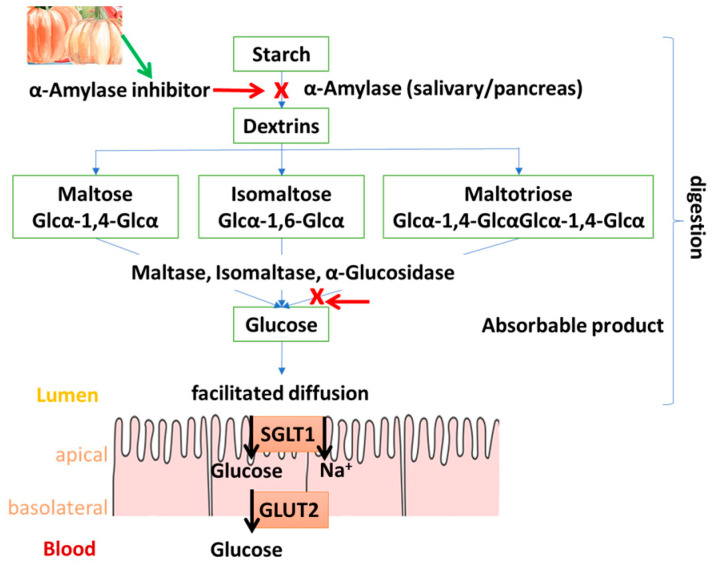
α-amylase and α-glucosidase inhibition pathways for hypoglycemic effects.

Among the digestive enzymes responsible for the breakdown of carbohydrates are α-amylase and α-glucosidase. The action of α-amylase is to digest complex carbohydrates by breaking them down into smaller molecules. α-glucosidase is involved in the breakdown of poli-, oligo-, and disaccharides, resulting in glucose molecules as the final product. These two enzymes are considered key in the absorption process in the intestines; hence, inhibitors of these enzymes can prevent postprandial hyperglycemia [18]. The inhibition of α-glucosidase and α-amylase is one of the strategies for controlling hyperglycemia. Complex carbohydrates reaching the small intestine cannot be directly absorbed by enterocytes of the epithelium. Firstly, they need to be degraded by α-amylase and α-glucosidase. Inhibitors of these enzymes slow the increase in glucose concentration in the small intestine and efficiently foster apical glucose transporters (GLUT2 and SGLT1) insertion, preventing an increase in blood glucose levels. Compounds that inhibit these enzymes could help lower the glycemic index of carbohydrate-rich foods, supporting the development of health-oriented products for diabetes management [112] (Figure 3).

Another enzyme that plays a role in glucose metabolism is dipeptidyl peptidase-IV (DPP-IV). The secretion of DPP-IV causes a decrease in glucose-like peptide-1 (GLP-1), which works by lowering blood glucose levels by supporting insulin secretion in hyperglycemia in patients with diabetes.

It is assumed that DPP-IV inhibitors can increase intestinal GLP-1 secretion, which in turn stimulates insulin secretion and helps maintain appropriate glycemia levels in patients with type 2 diabetes [18]. Several studies have been conducted using different parts of pumpkin in which the ability to inhibit α-amylase and α-glucosidase has been investigated. Studies using seed extracts (*C. maxima*), seed oils (*C. pepo*), and pumpkin leaf extracts (*C. pepo*) revealed an inhibitory effect on α-amylase and α-glucosidase. Additionally, Jane Monica et al. [89] have proven in an in vitro study that ethanol pumpkin seed extracts (*C. maxima*) also inhibit the DPP-IV enzyme, with the most effective extract at 500 mg/mL concentration inhibiting 81.20% of DPP-IV activity [18,89].

Watermelon pulp, seeds, and rind also exhibit antihyperglycemic activity and may positively influence the regeneration of pancreatic β cells, acting similarly to antidiabetic drugs. In the study by Sorour et al. [95] giving female Albino rats with induced type 2 diabetes a watermelon rind juice resulted in a reduction in blood glucose levels and alleviation of changes in the structure of the pancreas. In another study, the α-amylase and α-glucosidase inhibitory properties were assessed. The authors found that extracts from yellow flesh watermelon (seeds, flesh, rind, and leaf) effectively inhibited these two digestive enzymes [96].

Several studies are confirming the antidiabetic properties of kiwano. Busuioc et al. studied the potential antidiabetic activity of hydroethanolic extracts of *C. metuliferus*. In vitro test results confirmed the melon extracts’ inhibition of a-amylase and a-glucosidase, and the effect was compared to the drug acarbose. The study noted that the triterpenes and their derivatives present in kiwano can also positively affect glucose levels by inhibiting carbohydrate absorption. In addition, it is assumed that extracts from *C. metuliferus* may lower postprandial blood glucose levels by inhibiting the activity of β-glucosidase or α-amylase enzymes, which are involved in the digestion of complex carbohydrates taken in with food into monosaccharides that are absorbed in the intestine [113]. According to Chen and Kang [102], hexane extracts obtained from melon seeds also exhibit antidiabetic activity by inhibiting the enzymes α-amylase (61.8%) and α-glucosidase (35.3%) in an in vitro study. The authors of this study also examined the level of inhibition against these two digestive enzymes depending on the roasting seeds temperature (150 °C, 200 °C, 250 °C, and 300 °C), and the highest effectiveness was found at 250 °C (72.0%) [114].

According to the study conducted by Sharmin et al. [107], it has been proven that cucumber also has hypoglycemic properties. The authors of the study tested ethanol extracts of some fruits from the *Cucurbitaceae* family, including cucumber (*C. sativus*). The prepared extracts were given to rats with alloxan-induced diabetes. Based on the study, it was found that among the fruits tested, cucumber has the best hypoglycemic properties, the ethanol extract (200 mg/kg body weight) reduced glucose levels by 67% after 12 h of injection [114]. The authors suggest that several mechanisms cause fruit extracts, including cucumber extracts, to have a therapeutic effect. One of these mechanisms may be the stimulation of insulin action in plasma, which occurs because of insulin release by pancreatic β cells. Moreover, the therapeutic effect may involve increased peripheral glucose utilization as well as the intensification of glycolysis and glycogenosis processes with a simultaneous limitation of glycogenolysis and gluconeogenesis [107].

### 8.2. Hypolipidemic Effect and Cardiovascular Preventive Properties

Sh Ali [91] conducted a study in which they compared the lipid-lowering properties of cake with the addition of pumpkin extract in three variants (5, 10, and 15%) with a control cake made of wheat flour. The analysis of the in vivo study conducted on rats showed that pumpkin flour both lowers LDL cholesterol and increases HDL cholesterol, which correlates with the administered dose of pumpkin extract [107]. In another study, Gossell-Williams et al. [93] introduced pumpkin seed oil supplementation (2 g per day) for 12 weeks to postmenopausal women. The results of this study revealed that supplementation increased HDL cholesterol and decreased diastolic blood pressure [93]. With chronically elevated cholesterol levels, the endothelial layer becomes dysfunctional, resulting in an increased level of vascular cell adhesion molecules (VCAMs). The reason for this phenomenon is the activation of reactive oxygen species (ROS), which limit the formation of nitric oxide (NO) and thus increase the oxidation of LDL cholesterol. Pumpkin seed powders are a good source of arginine, an amino acid that is a precursor of NO [7]. A study conducted by Proboningsih et al. [94] revealed that the administration of pumpkin seed extracts to rats with hyperlipidemia leads to an increase in NO production due to the presence of the arginine. High NO concentration reduced LDL cholesterol oxidation and consequently reduced VCAM expression [7,94].

In an in vivo study conducted on mice by Poduri et al. [115], watermelon extracts were shown to have a positive effect on slowing down the development of atherosclerosis by lowering the concentration of cholesterol in blood plasma. Furthermore, it has been shown that L-citrulline, which is converted into L-arginine, has a vasodilating effect and thus improves blood flow through the vessels and prevents fat accumulation in cells [115]. Creatine kinase and lactate dehydrogenase are considered markers of myocardial damage. Karikpo et al.’s [97] study investigated the cardioprotective properties of watermelon seeds’ ethanolic extracts. The results of the conducted studies revealed that the administration of watermelon seed extracts to albino rats with streptozotocin-induced diabetes caused a decrease in creatine kinase and lactate dehydrogenase depending on the dose and duration of treatment. The decrease in the activity of these two enzymes after supplementation with *C. lanatus* extracts suggests a significant cardioprotective potential to improve the structural integrity of the myocardium in the diabetic state [97]. Another study examined the cardioprotective properties of watermelon rinds. The research results revealed that introducing 10% watermelon rinds into the diet of rats with hypercholesterolemia resulted in a significant reduction in total cholesterol from 266.2 to 222.7 mg/dL and LDL cholesterol from 159.5 to 94.4 mg/dl in blood serum [116]. Carillon et al. [106] studied the cardioprotective properties of melon (*Cucumis melo* L.) concentrates. A 4-week supplementation introduced in hamsters with induced obesity resulted in the alleviation of vasoconstrictive dysfunction associated with morphological remodeling. The positive change was caused by increased NO bioavailability and endogenous superoxide dismutase (SOD) expression [106]. Kiwano seeds may exhibit cardioprotective potential, which is attributed to the presence of two acids in these seeds: linoleic and oleic. These compounds show strong properties related to lowering LDL cholesterol and thus preventing heart attacks and strokes [43]. In one of the randomized, double-blind studies, the effect of cucumber (*C. sativus*) seed extract on the level of lipids in the blood serum in adult patients with abnormal lipid profile parameters was examined. Six weeks of supplementation in patients receiving cucumber seed extract resulted in a significant decrease in total cholesterol, LDL cholesterol, and TG. Moreover, a significant increase in HDL cholesterol was observed compared to the placebo group [108].

### 8.3. Antioxidant Effect

Chen et al. [88] investigated the biological activity of pumpkin polysaccharides. The antioxidant activity of polysaccharides is assessed by the ability to scavenge free radicals, including anion radicals (O^2−•^), hydroxyl radicals (•OH), and their active derivatives (such as H_2_O_2_). The results of the in vitro study revealed that pumpkin polysaccharides increased, among others, the activity of glutathione peroxidase (GSH-Px) and superoxide dismutase (SOD) in the serum, spleen, and kidney tissue of mice. These enzymes (GSH-Px and SOD) form part of the body’s antioxidant defense system, working to prevent oxidative damage to cells and tissues [88]. Pumpkin seeds are characterized by a high content of compounds with antioxidant activity, including *β*-carotene and tocopherols. These compounds have a protective effect on cells, preventing them from being damaged by reactive oxygen species (ROS) and other free radicals [6].

Phenolic compounds, carotenoids, and unsaturated fatty acids are the main components found in watermelon, which exhibit antioxidant activity. Oxidative stress is believed to be responsible for the development of some chronic diseases [39]. Lycopene, which is a compound found in large quantities, especially in watermelons with red flesh, has a mitigating effect on oxidative stress [98]. Additionally, the antioxidant activity of watermelon was assessed in vivo by measuring the enzymatic activity of enzymes that protect cells from ROS, i.e., superoxide dismutase (SOD), catalase (CAT), glutathione peroxidase (GPx), and glutathione S-transferase (GST). The results of the study confirmed that watermelon consumption correlates with greater antioxidant capacity and a reduction in oxidative stress [39,100]. Feizy et al. [100] showed in vitro that watermelon peel indicates a notable DPPH free radical scavenging activity with an IC_50_ = 147.30 mg/kg. Wen et al. [99] investigated the antioxidant activity of peptides obtained from watermelon seed protein hydrolysates in an in vitro study. The peptides tested showed antioxidant activity by protecting HepG2 cells from H_2_O_2_-induced oxidative damage and increasing the activity of antioxidant enzymes [99]. Various studies have been conducted to confirm the antioxidant properties of kiwano, including both its edible parts (flesh) and inedible parts (seeds and peel). Bolek et al. demonstrated the presence of nutritional compounds and phytochemicals with the ability to scavenge free radicals. Additionally, methanolic extracts from horned melon exhibited DPPH and ABTS radical scavenging activities of 20.88 and 185.36 μmol Trolox equivalent per gram, respectively, in an in vitro assay [103].

Melon fruits owe their antioxidant activity primarily to the presence of polyphenols and carotenoids [117]. A study conducted on extracts from different parts of melon, i.e., pulp, seeds, and peels, revealed that extracts from melon peels showed the highest antioxidant activity [104]. In one study, the 2,2-diphenyl-1-picrylhydrazyl (DPPH^•^) and hydroxyl radical scavenging activity of methanol extracts obtained from different parts (skin, leaf, stem, flesh, and seeds) of cantaloupe melon was determined. The highest IC_50_ value was found for the melon seed extract 25.44 ± 2.83 mg/mL for the DPPH^•^ inhibition test, while for the hydroxyl radical scavenging activity, the highest value was 147.96 ± 22.04 g dimethyl sulfoxide (DMSO) equivalents/g extract for melon leaf indicating strong antioxidant potential in vitro [104].

The study carried out by Yunusa et al. [49] showed that different parts of cucumber exhibit different antioxidant activity. In this in vitro study, DPPH radical scavenging activity ranged from 6.61% for an aqueous extract of the flesh to 20.18% for an ethanol extract of the whole cucumber fruit. In the same study, ferric-reducing antioxidant power (FRAP) was examined. The highest result was recorded for the ethanol extract of the peel (0.12 mmol Fe^2+^/g), and this value was significantly higher than that of extracts from other parts of the cucumber [49]. Other researchers have also confirmed through in vitro studies that cucumber pulp is a strong antioxidant with an IC_50_ value of 14.73 ± 1.42 µg/mL, while for cucumber leaves, the IC_50_ value is 13.06 µg/mL [109,110].

### 8.4. Anticancer Effect

Although progress has been noted in cancer treatment in recent years, implementing an appropriate treatment strategy is still very difficult, and most cancers are completely incurable. Difficulties in finding an effective method of combating cancer cells result primarily from the ability of cancer cells to infinite replication, as well as diffusional transfer [118]. Carotenoid-enriched pumpkin extracts (*C. moschata*) were shown to retard cell proliferation in a human chronic lymphocytic leukemia cell line by modulating autophagic flux [12,90]. Compounds found in pumpkin peel such as cucurmosin, low-starch polysaccharides, and lignin also exhibit anticancer activity [12]. Pumpkin seed oil has also been proven to be beneficial in the case of benign prostatic hyperplasia, as well as in the early stages of prostate cancer, based on both animal (rat) and human studies [7]. Moreover, hydroethanolic extract from pumpkin seeds (*C. pepo*) has been shown to have a beneficial effect on reducing the viability of breast (MCF-7 (ERα positive)) and colon adenocarcinoma (Caco-2) cell lines [3,92]. Methanol extracts obtained from watermelon seeds have shown beneficial effects in reducing benign prostatic hyperplasia. As a result of a month of administration of methanol extracts from watermelon seeds to rats, the prostate was significantly reduced, but the original size of the shrunken testicles was not restored, and advanced oligospermia caused by hormones was not reversed [119]. Furthermore, Dammak et al. [101] demonstrated that watermelon peel extracts exhibited cytotoxic activity against Hep-2 (a human epithelial type-2 cell) in a time- and dose-dependent manner.

One of the stronger bioactive compounds that can potentially be used in the therapy of certain cancers is cucurbitacin. In one of the studies using methanolic extracts of melon, the compound cucumol A (a compound belonging to the cucurbitacin group) was identified. This bioactive molecule exhibits in vitro cytotoxic activity against cancer cells L5178Y and HeLa (mouse lymphoma cell line and human cervical cancer cell line, respectively) [105]. Furthermore, cucurbitacin B, which is also found in melon, is considered a natural anticancer compound due to its ability to inhibit the proliferation of various leukemia cells, usually by inhibiting STAT3 activation and the Raf/MEK/ERK pathway [120]. Sadou et al. examined the antioxidant profile of horned melon seed oil. The analysis revealed high levels of α and γ-tocopherols, compounds whose consumption is considered a cancer-preventive factor [121].

A study using acetyl extracts from cucumber flowers showed that the anticancer activity directed against HePG2 cells is mainly based on the high content of bioactive compounds, including cucurbitacins, lignins, and flavonoids [9]. Sharma et al. [111] investigated the potential of a vitexin derived from cucumber as an antiangiogenic agent through molecular docking analysis. They found that it exhibited a significant inhibitory effect on the Hsp90 protein, which plays a key role in indirectly promoting angiogenesis and metastasis [111].

## 9. Conclusions

Cucurbitaceae plants are a rich source of nutrients, minerals, and bioactive compounds, which makes them high-value dietary components with remarkable health advantages. Their low caloric value, glycemic index, and glycemic load further enhance their relevance in the treatment of metabolic disorders such as obesity, insulin resistance, and diabetes. Moreover, certain species within the cucurbit family exhibit distinct functional properties, making them beneficial for targeted health applications.

For instance, *Cucurbita pepo* (pumpkin) possesses a wide range of therapeutic properties, including diuretic, anti-inflammatory, hepatoprotective, antioxidant, and hypoglycemic effects, while its seeds are specifically known for their hypolipidemic activity. *Citrullus lanatus* (watermelon) is highly valued for its antioxidant and diuretic effects, while its seeds have antimicrobial, hepatoprotective, cardioprotective, and anti-inflammatory properties. *Cucumis melo* (melon) promotes skin hydration and exerts antioxidant and anti-inflammatory effects, while its peel and seeds show antiproliferative potential. *Cucumis metuliferus* (horned melon, kiwano) shows antioxidant and cardioprotective properties and is also used in the treatment of gastrointestinal diseases. *Cucumis sativus* (cucumber) is noted for its hypolipidemic, antiaging, and antimicrobial properties.

The wide spectrum of biological properties of cucurbit plants creates the possibility of their application not only in the medical and food sectors but also in cosmetics and pharmaceutical industries. Their antioxidant, antiaging, and antimicrobial activities render them promising ingredients in skincare and personal care products, while their antimicrobial and antiparasitic properties are likely to be significant in natural preservative formulations.

The potential of bioactive compounds derived from plants of the *Cucurbitaceae* family and their application in the creation of functional foods dedicated to specific health benefits should be the direction of future scientific research. Effective encapsulation necessary to maintain the bioavailability and activity of these compounds in the human body is a key challenge for food technology. Progress in methods of encapsulating bioactive compounds and their effect on metabolism can enhance dietary strategies supporting health and preventing diseases.

## Figures and Tables

**Table 1:** The nutritional composition of selected Cucurbitaceae family plants.

Components (%)	Pumpkin (*C. moschata, C. pepo, C. maxima*)					Watermelon (*C. lantus, C. vulgaris*)			Melon (*C. melo*)			Horned Melon (*C. metuliferus*)			Cucumber (*C. sativus*)			References
	Flesh	Peel	Seeds	Flowers (Various Species)	Leaves (*T. occidentalis*)	Flesh	Peel	Seeds	Flesh	Peel	Seeds	Flesh	Peel	Seeds	Flesh	Peel	Seeds	
**Carbohydrate**	2.62–48.40	4.37–20.68	6.37–37.9	3.02–3.90	26.82	3.10–8.00	42–65	6.06–21.20	0.50–1.31	3.05–57.90	8.00–39.00	7.56	54.80	50.2	52.70	33.70	50.10	[4,8,9,12,15,16,17,19,20]
**Protein**	0.20–15.50	0.92–23.95	14.31–38.0	1.14–1.50	56.00	0.12–0.60	6.77	34.00–35.00	3.24	0.80–3.05	14.91–29.90	1.78–1.80	2.95	2.63	15.90	26.50	38.88	
**Lipid**	0.04–0.42	0.31–6.57	21.9–54.9	0.21–0.33	2.00	0.05–0.10	0.92	26.83–46.78	1.10	1.58–2.12	35.60–47.00	0.03–1.26	8.89	15.4	0.13	1.44	95.79	
**Fiber**	0.37–11.25	0.13–33.92	1.00–16.15	-	-	0.20–0.60	24	2.00–3.00	8.83	41.69	5.51–24.75	4–4.20	11.30	19.2	6.77	8.86	3.00	
**Ash**	0.34–6.64	0.63–10.65	3.0–5.50	0.85–1.92	7.73	0.28–1.13	3.07–13.20	3.00	2.40	3.67	1.50–4.83	-	-	-	11.60	7.85	9.34	
**Moisture**	18.03–96.77	9.76–93.59	1.80–7.40	-	7.45	90.82–95.00	95.63	8.00–9.00	83.05	16.95	4.27–7.78	89–96.0	18.4	7.31	12.90	21.70	12.30	

**Table 2:** The content of macro- and microelements of selected *Cucurbitaceae* family plants.

Mineral Composition	Pumpkin (*C. moschata, C. pepo, C. maxima*) (mg/100 g)					Watermelon (*C. lantus, C. vulgaris*)			Melon (*C. melo*) (mg/100 g)			Horned Melon (*C. metuliferus*) (mg/100 g)			Cucumber (*C. sativus*) (mg/100 g)			References
	Flesh	Peel	Seeds	Flowers (Various Species and Varieties)	Leaves (*T. occidentalis*)	Flesh (mg/g)	Peel (mg/g)	Seeds (mg/100 g)	Flesh	Peel	Seeds	Flesh	Peel	Seeds	Flesh	Peel	Seeds	
**Calcium**	21	1.360	8.44–141.00	13.78–37.77	75.0	1.87	3.10	0.16	855.25	14.69–4201.4	8.34–806.4	13–17	-	247	139	168	177	[6,7,12,16,17,19,20,28]
**Iron**	0.8	4.004	3.75–1676	1.26–5.29	3.7	0.34	0.22	3.71	1.82	0.4–3.4	2.69–81.17	0.50–1.13	-	10.90	7.80	7.39	9.08	
**Magnesium**	12	3.353	67.41–2385.00	17.72–35.83	33.0	1.85	2.72	0.15	328.75	13.27–389.65	101.71–3299.27	16.2–40	-	289	70.8	64.1	64.4	
**Phosphorous**	44	1.419	47.68–1471.24	-	-	4.64	7.81	0.17	-	-	-	37–50	-	44.70	-	-	-	
**Potassium**	340	687.47	103.12–4300.00	199.73–349.66	352.0	18.53	88.67	3.57	2113.75	110.39–1791.9	509.8–9548.33	123–302	-	1174	437	454	541	
**Sodium**	1.0	9,652	0.69–189.81	-	89.7	-	-	-	137.58	8.54–277.9	41.22–386.13	2–5.60	-	247	151	113	156	
**Zinc**	0.32	0.150	1.09–14.14	0.48–0.78	19.3	0.038	0.045	3.71	0.7	0.23–2.3	2.34–44.03	0.20–0.48	-	1.70	5.27	2.66	5.46	
**Copper**	0.127	0.025	0.30–89.84	-	36.4	0.028	0.005	0.38	0.2	0.07–8.9	0.53–15.9	0.10	-	5.40	2.49	1.69	2.21	
**Manganese**	0.125	0.360	0.06–8.90	0.18–0.38	-	0.057	0.095	0.02	0.48	0.41–4.1	1.25–15.20	0.039	-	-	0.79	0.4	0.56	
**Selenium**	0.3 µg	-	0.13–1.91	-	-	-	-	-	-	-	-	13–17	-	247	-	-	-	

## Data Availability

No new data were created or analyzed in this study. Data sharing is not applicable to this article.

## Abbreviations

The following abbreviations are used in this manuscript:

Caco-2	colon adenocarcinoma
CAE	chlorogenic acid equivalent
CAT	catalase
CE	catechin equivalent
DMSO	dimethyl sulfoxide
DPPH	2,2-diphenyl-1-picrylhydrazyl
DPP-IV	dipeptidyl peptidase-IV
FRAP	ferric-reducing antioxidant power
GAE	gallic acid equivalent
GI	glycemic index
GL	glycemic load
GLP-1	glucose-like peptide-1
GLUT2	glucose transporter-2
GSH-Px	glutathione peroxidase
GST	glutathione S-transferase
HDL	high density lipoprotein
Hep-2	human epithelial type-2 cell
LDL	low-density lipoprotein
NO	nitric oxide
QE	quercetin equivalent
ROS	reactive oxygen species
SGLT1	sodium glucose cotransporter-1
SOD	superoxide dismutase
TAE	tannic acid equivalent
TFC	total flavonoids content
TG	triglyceride
TPC	total phenolic content
VCAM	vascular cell adhesion molecule
